# Influence of gravity compensation training on synergistic movement patterns of the upper extremity after stroke, a pilot study

**DOI:** 10.1186/1743-0003-9-44

**Published:** 2012-07-23

**Authors:** Thijs Krabben, Gerdienke B Prange, Birgit I Molier, Arno HA Stienen, Michiel JA Jannink, Jaap H Buurke, Johan S Rietman

**Affiliations:** 1 Roessingh Research and Development, Roessinghsbleekweg 33B, Enschede, the Netherlands; 2 Department of Biomechanical Engineering, University of Twente, Drienerlolaan 5, Enschede, the Netherlands; 3 Rehabilitation Centre 'het Roessingh', Roessinghsbleekweg 33, Enschede, the Netherlands

**Keywords:** Stroke, Synergy, Gravity compensation, Upper extremity, Circle drawing, Reach training

## Abstract

**Background:**

The majority of stroke patients have to cope with impaired arm function. Gravity compensation of the arm instantaneously affects abnormal synergistic movement patterns. The goal of the present study is to examine whether gravity compensated training improves unsupported arm function.

**Methods:**

Seven chronic stroke patients received 18 half-hour sessions of gravity compensated reach training, in a period of six weeks. During training a motivating computer game was played. Before and after training arm function was assessed with the Fugl-Meyer assessment and a standardized, unsupported circle drawing task. Synergistic movement patterns were identified based on concurrent changes in shoulder elevation and elbow flexion/extension angles.

**Results:**

Median increase of Fugl-Meyer scores was 3 points after training. The training led to significantly increased work area of the hemiparetic arm, as indicated by the normalized circle area. Roundness of the drawn circles and the occurrence of synergistic movement patterns remained similar after the training.

**Conclusions:**

A decreased strength of involuntary coupling might contribute to the increased arm function after training. More research is needed to study working mechanisms involved in post stroke rehabilitation training. The used training setup is simple and affordable and is therefore suitable to use in clinical settings.

## Background

### Introduction

Stroke is one of the main causes of disability in Europe [1] and North America [2]. Due to hemorrhagic or ischemic damage to brain tissue, motor planning and the integration of sensorimotor information are degraded. In many cases, this results in an altered generation of muscle activity, which may present as weakness, co-contraction and disturbed timing [3,4]. Coordination between muscles can also be impaired, leading to less selective movements. In clinical practice, stereotypical movement patterns because of abnormal muscle synergies are often observed [5,6]. Movements are restrained within either a flexion synergy (shoulder abduction, shoulder external rotation, elbow flexion and forearm supination) or an extension synergy (shoulder adduction, shoulder internal rotation, elbow extension and forearm pronation), or a combination of components of both synergies [7]. In the majority of stroke patients, these limitations account for a reduced ability to use the arm. During rehabilitation training, restoration of (partly) lost functions is stimulated and compensational strategies are promoted in order to increase the functional abilities of the affected arm and increase the level of independence. At most 20% of all patients have complete arm function 6 months post stroke [8].

### Synergies

In stroke patients, abnormal coupling between shoulder and elbow movements was observed during isometric contractions: high torques of shoulder abduction are related to simultaneous elbow flexion torques [9,10]. Indications for coupling of these components were also observed in muscle activity during isometric contractions [11]. In the case of reaching movements, a certain amount of shoulder abduction is needed to lift the arm, provoking simultaneous elbow flexion torques and limiting elbow extension [12,13].

### Gravity compensation

A way to instantaneously reduce the influence of these abnormal, post stroke synergistic patterns (i.e. abnormal coupling) is to counterbalance the weight of the arm. As recent research has shown, arm support decreases the required shoulder abduction torques during two-dimensional reaching movements at shoulder height, subsequently causing a decrease in coupled elbow flexion, leading to an increase in the range of elbow extension [12,13]. Using the gravity compensation device ‘Freebal’ [14], similar results were found in a study examining maximal reaching distance during supported and unsupported three-dimensional reaching movements of stroke patients [15]. Regarding muscle activity, research in healthy persons showed that the application of gravity compensation facilitates movements by instantaneously reducing the amount of muscle activity needed for a reaching movement, particularly in muscles counteracting gravity [16]. Similar results were observed in a sample of chronic stroke patients with mild hemiparesis [17].

The facilitating influence of gravity compensation can be used to improve unsupported arm movements in stroke patients. Since gravity compensation has shown to instantaneously reduce the influence of abnormal synergies in cross-sectional studies, one can hypothesize that a long(er) term application has the potential to reduce the degree in which abnormal synergies affect unsupported arm movements of stroke patients. Several studies have shown that reach training using arm support can result in improved movement ability of the unsupported hemiparetic arm. After arm training using a passive exoskeleton to support the arm, motor status of the arm improved [18,19]. This improvement was accompanied by an increased maximal reaching distance [18]. A training period with sling suspension also induced a modest improvement in motor status of the arm [20]. Although maximum reaching distance increased, little is known about the underlying mechanisms causing these beneficial results. It is still unclear whether a reduction of the impact of abnormal coupling is involved in those improvements of arm function.

Previous studies showed that abnormal coupling influences circle drawing performance. Due to synergistic movement constraints, elliptical instead of round shapes are produced by stroke patients during supported [21] and unsupported [22] circle drawing. After a period of robot-aided point-to-point arm movement training in a gravity-compensated environment, elliptical shapes drawn by a sample of 117 stroke subjects changed towards circular movements. Changes in supported circle drawing were due to reduced impact of abnormal coupling and a consequently more selective, or more isolated, control of shoulder and elbow movements [21].

### Objective

Therefore, the objective of the present study is to examine whether a long(er) term application of gravity compensation affects the influence of abnormal synergies on unsupported arm movements in a sample of chronic stroke patients, using circle drawing performance to identify synergistic movement patterns. This explorative research may increase the understanding of the effect of gravity compensation training on unsupported arm movements by providing a better insight into the role of abnormal coupling in training-induced changes of arm movement ability.

## Methods

### Subjects

Subjects were recruited at rehabilitation centre ‘Het Roessingh’ (RCR) in Enschede, the Netherlands. Inclusion criteria were: 1) a history of a single unilateral stroke in the left hemisphere, resulting in a right-sided hemiparesis, 2) the onset of the stroke was more than six months (chronic phase) before the start of the intervention period, 3) ability to move the shoulder and elbow joint against gravity but unable to hold the joints against a combination of moderate resistance and gravity, and 4) adequate cognitive function to understand the experiments, follow instructions, and give feedback to the researchers. Subjects were excluded from this study if: 1) a fixed contracture deformity in the affected upper limb was present, 2) pain was a limiting factor for the subject's active range of motion, or 3) if they participated in other training experiments. All subjects provided written informed consent. The study was approved by the local medical ethics committee.

### Gravity compensation training

Subjects received three half-hour gravity compensation training sessions per week for a period of six weeks, making a total of 18 sessions. To study the effect of gravity compensated rehabilitation training two baseline and one evaluation measurement were performed. Baseline measurements were performed two weeks prior to the start of the intervention period, spaced one week apart. Within one week after the last training session, subjects performed the evaluation measurement.

**Figure 1:**
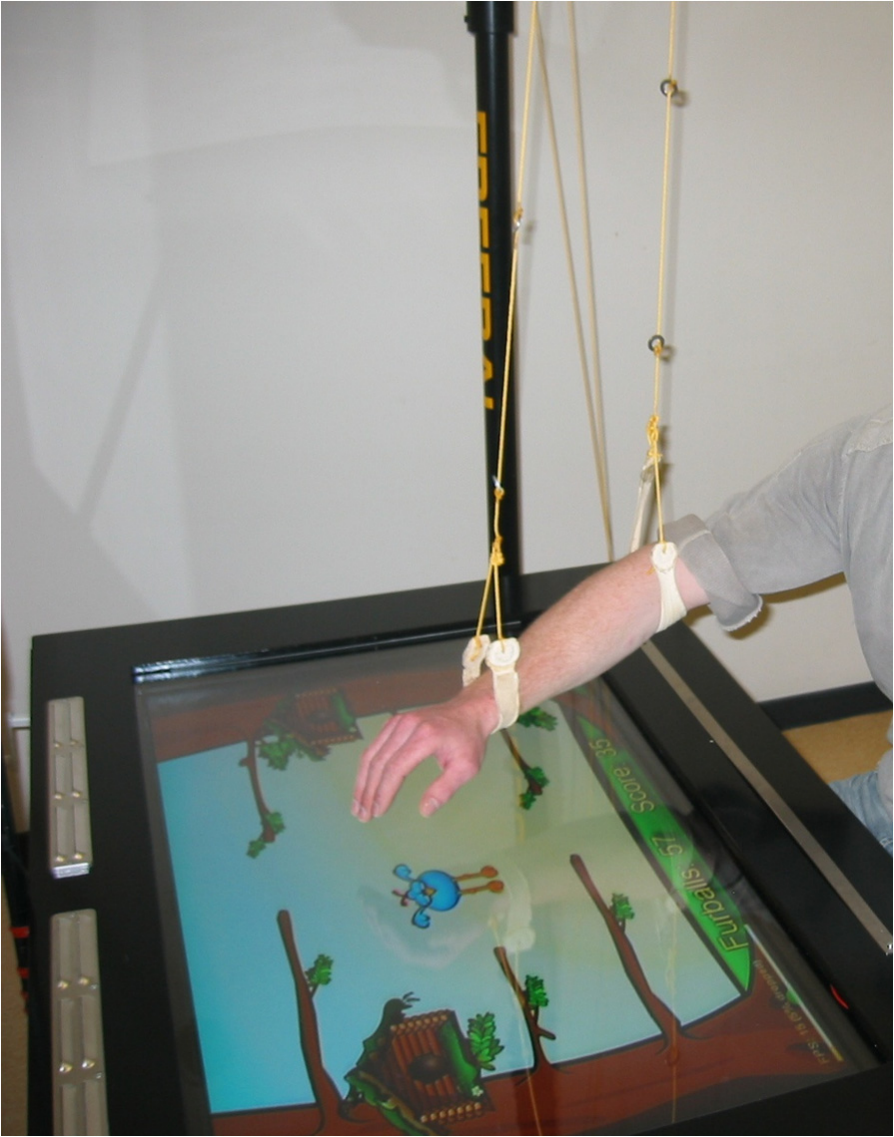
Training setup with FurballHunt and Freebal.

During a training session subjects practiced three-dimensional (3D), goal-directed arm movements in a gravity compensated, virtual reality (VR) augmented environment (see Figure 1). The weight of the subject’s arm was (partially) counterbalanced by a gravity compensation system device named Freebal [14], providing a constant amount of support during natural 3D movements. The Freebal consists of two overhead slings connected to ideal spring mechanisms by wires. One sling supports the subject’s arm at the elbow joint and one sling at the wrist. The Freebal allows easy and quick adjustment of the level of gravity compensation by altering the force which is applied to the slings by the spring mechanisms. If arm function improved, indicated by increasing scores of the FurballHunt game throughout the training, the supervising physical therapist decreased the level of gravity compensation to ensure a challenging and motivating training environment.

Virtual reality was delivered by a game named FurballHunt [23], in which the user has to chase away little birds, called Furballs [23]. During the game, Furballs fly from a birdhouse to a tree branch where they sit down, while the user holds the affected hand on a start button. The starting position of the user is with the upper arm along the trunk and the elbow bent approximately 90 degrees, with the hand on the start button at table height. The bird can be chased away by lifting the hand from the start button and moving the hand towards the bird, i.e. reaching forward at table height, and touch it. Motion capturing software detects arm movement by substraction of two consecutive images obtained from a commercially available webcam (Logitech Quickcam Messenger, Logitech Inc., Fremont, CA, USA) that is located above and aiming towards the screen that displays FurballHunt. Points are awarded to the user if a Furball is chased away within a certain time frame. The game was shown on a horizontally placed flat screen, which is mounted on an in height adjustable frame, see Figure 1. All training sessions were supervised by the same physical therapist, who decided when the difficulty level had to be increased, based on clinical experience. The level of gravity compensation was decreased with steps of approximately 10% when maximal scores of the FurballHunt game were approached. Throughout the training, reaching distance (i.e. location of the tree branches), training intensity (i.e. the number of Furballs) and the level of randomization of target sequence were increased, to maintain a challenging training environment for each user. The level of gravity compensation, the number of performed reaching movements and the level of target randomization were stored in a logbook by the trained physical therapist.

### Procedures

All measurements were performed by one researcher, who was not involved in the training sessions. During evaluation measurements subjects performed an unsupported, i.e. without gravity compensation, circle drawing task at table height. Before movement execution, upper and forearm lengths were measured. Upper arm length was measured between the ventral tip of the acromion and the lateral epicondyle of the humerus. Forearm length was measured between the lateral epicondyle of the humerus and the third metacarpophalangeal joint. After measurement of the arm lengths, a non-actuated instrumented exoskeleton (Dampace [24]) to measure joint angles was attached to the upper and forearm and the wrist was immobilized with a splint. To minimize trunk and shoulder movement, subjects were strapped with a four point safety belt. Subjects were asked to perform a circular motion in the transversal plane, just above a tabletop, in a clockwise (CW) and counter-clockwise (CCW) direction. The order of direction has been randomized throughout the measurements. Subjects were instructed to draw five circles in each direction, as big and as round as possible. For the latter purpose template circles of different radii were shown on a tabletop. Movements were performed at a self selected speed while verbal encouragement was provided to the subjects throughout the experiment.

### Measurements

During evaluation measurements, the upper extremity part of the Fugl-Meyer (FM) was assessed to clinically evaluate arm function. Joint angles of the shoulder and elbow were recorded with the instrumented exoskeleton [24]. Built-in potentiometers on three rotational axis of the shoulder joint allow measurements of upper arm elevation, transversal rotation, and axial rotation. Elbow flexion and extension were measured with a rotational optical encoder. Translations of the shoulder were measured with linear optical encoders. Signals from the potentiometers were converted from analog to digital (AD) values by a 16 bits AD-converter (PCI 6034, National Instruments, Austin, Texas). The rotational and linear optical quadrature encoders were sampled by a 32 bits counter card (PCI6602, National Instruments, Austin, Texas). Digital values were sampled with a sample frequency of 1 kHz, on-line low-pass filtered with a first order Butterworth filter with a cut-off frequency of 40 Hz and stored on a computer with a sample frequency of 50 Hz.

### Data analysis

Because the focus of the present study is on proximal arm function, a subset of the upper extremity part of the FM scale consisting of items A_II_, A_III_ and A_IV_ (max. 30 points) that reflect the ability to move the shoulder, elbow and forearm within and out of pathological synergies was addressed separately (FMp). Positions of the shoulder, elbow, and hand were calculated from the measured joint angles and arm lengths. To exclude contributions of shoulder and trunk movements to the size of the circles drawn by the subjects, the position of the hand relative to the position of the shoulder was calculated.

Active work area of the arm was represented by the area of the circles that was calculated as the area enclosed by the projection of the hand trajectory onto the table surface. The three largest circles in both directions (CW and CCW) were selected for further analysis. To compensate for differences in arm length among subjects, circle area is normalized (normA) to arm length by dividing circle area by the maximal circle area that is biomechanically possible. Circle area is considered maximal when the diameter of the circle equals the arm length of the subject.

**Figure 2:**
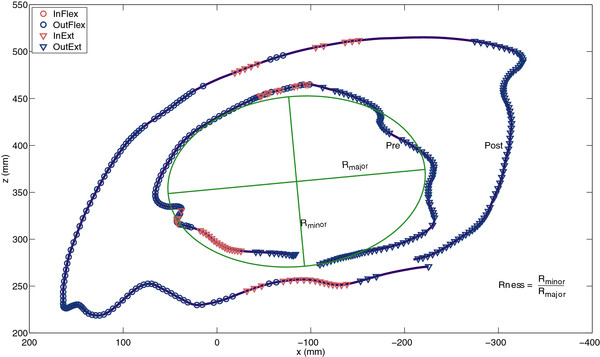
** Typical example of circles drawn before (Pre) and after (Post) training.** Roundness (Rness) is calculated as the quotient of the length of the minor axis (Rminor) and the major axis (Rmajor) of the fitted ellipse (green).

A method [25] to calculate circle roundness was used to quantify circle morphology. In this method, circle roundness is defined as the quotient of the minor and major axes of a fitted ellipse, see Figure 2 for a graphical representation. This method was previously used to quantify preferred movement directions and circle roundness to evaluate gravity compensated reach training in a sample of chronic stroke survivors [21,26].

**Figure 3:**
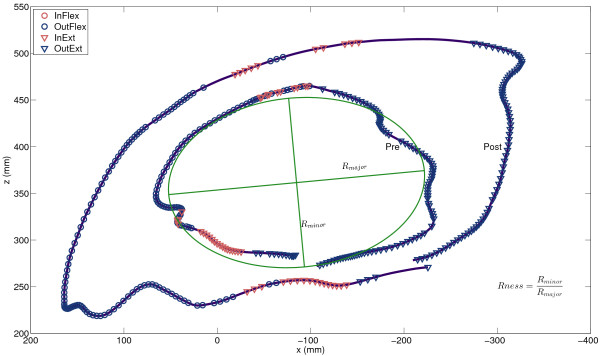
** Visual representation of the joint angles of the upper arm.** Arrows indicate positive rotations. EP = Elevation Plane, EA = Elevation Angle, AR = Axial rotation, EF = Elbow Flexion.

Thoracohumeral joint angles were calculated from the measured joint angles according to the recommendations of the International Society of Biomechanics [27]. Orientation of the upper arm was represented by the plane of elevation (EP), elevation angle (EA) and axial rotation (AR), see Figure 3. Joint angles were offline filtered with a zero-phase shift, 2^nd^ order Butterworth low-pass filter with a cut-off frequency of 10 Hz. Joint excursions were calculated as the range of each measured joint angle needed to draw one circle. To study the potential effect of gravity compensation training on elbow flexion and extension (EF) in more detail, maximal and minimal values of EF were calculated, besides the excursion.

To study the potential role of abnormal synergies during circle drawing tasks, circles were divided into four combinations of shoulder abduction/adduction and elbow flexion/extension. Shoulder abduction/adduction was defined as decrease/increase of the elevation angle in the plane of elevation, as recommended by the International Society of Biomechanics [27], also see Figure 3. Flexion and extension synergies were identified, based on simultaneous changes in EA and EF according to the method described in [22]. Movement within the flexion synergy (InFlex) is defined as simultaneous shoulder abduction (EA ↓) and elbow flexion (EF ↑). Other synergistic patterns were defined in a similar way: movement within the extension synergy (InExt) is characterized by simultaneous shoulder adduction (EA ↑) and elbow extension (EF ↓); concurrent shoulder abduction (EA ↓) and elbow extension (EF ↓) represents movement out of the flexion synergy (OutFlex); shoulder adduction (EA ↑) and elbow flexion (EF ↑) indicate the ability to move out of the extension synergy (OutExt). All combinations were calculated as percentages of movement time. The remaining part indicates to which extent subjects performed single joint movements (SJMov). InFlex and InExt represented movement within a synergistic pattern (InSyn). The ability to move out of a synergistic pattern (OutSyn) was calculated as the sum of OutFlex and OutExt. See Figure 2 for typical examples of circles drawn before and after training, and the occurrences of synergistic movement patterns.

### Statistical analysis

Consistency of the data obtained during both baseline measurements was evaluated by calculation of the intraclass correlation coefficient (ICC) according a two-way mixed model. Outcome measures obtained during both baseline measurements were statistically tested by means of a Wilcoxon signed rank test to reveal a possible learning effect between both measurements. These initial analyses revealed some variation (ICC ≥ 0.43) in motor performance, but differences were not statistically significant (p ≥ 0.09) and no clear trend was visible. Since an equal number of datapoints is needed for pairwise comparison of outcome measures before and after training, data of both baseline measurements were averaged per subject and compared with the data obtained during the evaluation measurement. Data in the results section are reported as median and interquartile (25^th^ - 75^th^ percentile) range (IQR). Because of the small sample size, training effects were non-parametrically tested for significance by means of a related samples Wilcoxon signed ranks test. Spearman’s correlation coefficients between outcome measures were calculated. Effects were considered statistically significant for p<0.05.

## Results

### Subjects

A convenient sample of 59 patients who have received treatment at RCR were screened. From this group 22 were contacted. A total of 12 patients did not meet the inclusion criteria because of a fully recovered arm (n = 3), an a-functional arm (n = 1) or refused to participate because of time constraints (n = 7) and too high travelling costs (n = 1). Initially ten subjects participated in this study. One subject (S3) withdrew after two weeks of training because of a too high physical burden, mainly caused by the distance he had to cover travelling from his home to the rehabilitation centre. One subject (S6) had a cerebellar infarction while the other subjects experienced a first-ever ischemic stroke in the medial cerebral arteric region. One subject (S10) was not able to complete the evaluation tasks because of physical limitations. Data from these subjects were excluded from further analysis for reasons of incompleteness (S3 and S10) and heterogenity (S6). Demographic data at baseline of the remaining seven subjects are displayed in Table 1.

**Table 1:** Subject demographic and clinical characteristics at baseline

***Subject***	***Gender***	***Dominance***^*§*^	***Months post-stroke***	pre FM*	pre FMp*
1	M	Right	58	12.0	6.0
2	F	Right	13	45.5	22.0
4	F	Right	27	10.0	4.0
5	F	Right	24	44.5	18.0
7	F	Right	39	45.5	17.5
8	F	Right	39	7.0	1.5
9	M	Right	8	25.5	13.5
Group^‡^	-		27 (15.8 – 39.0)	25.5 (10.5 – 42.3)	13.5 (4.5 – 17.9)

* Averaged value of two baseline measurements.

^§^ Before stroke.

^‡^ Median (25^th^ -75^th^ percentile).

*FM* = Fugl-Meyer, *FMp* = Proximal part of the Fugl-Meyer score (max 30).

### Gravity compensation training

**Figure 4:**
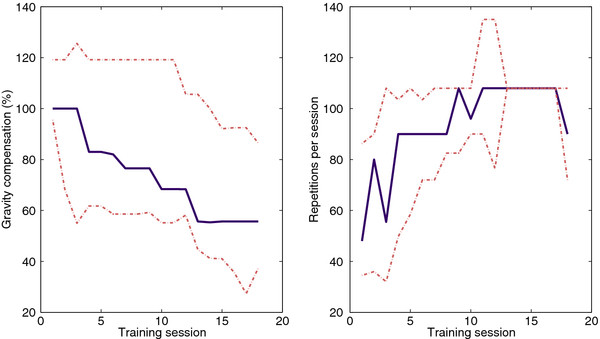
** Level of gravity compensation in % of the arm weight (left) and training intensity (right) throughout the training.** Blue solid lines are median values. Red dashed lines indicate 25th and 75th percentile.

The level of gravity compensation and the training intensity throughout the training are graphically displayed in Figure 4. Two severely affected subjects (S8 and S9) were overcompensated at the beginning of the training. The level of gravity compensation decreased throughout the training in all subjects. The number of reaching movements per session increased in all subjects throughout the training.

### Clinical evaluation

**Figure 5:**
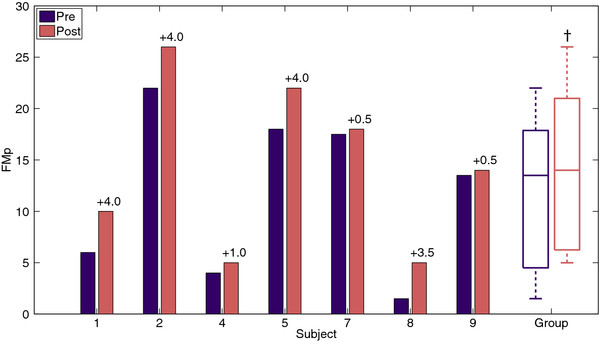
** Scores of the proximal part of the upper extremity part of the Fugl-Meyer assessment (FMp) per subject and group before (Pre) and after (Post) training.** Changes in FMp are displayed above the bars. † Indicates a statistically significant change.

FMp scores before and after the training are graphically displayed in Figure 5. On group level a statistically significant (p = 0.017) median increase of 3.5 points on the FMp scale is noticed after six weeks of training. Four subjects showed an increase of more than 10 percent of the maximal value, i.e. 3 points, which is considered clinically relevant [28].

### Circle metrics

**Figure 6:**
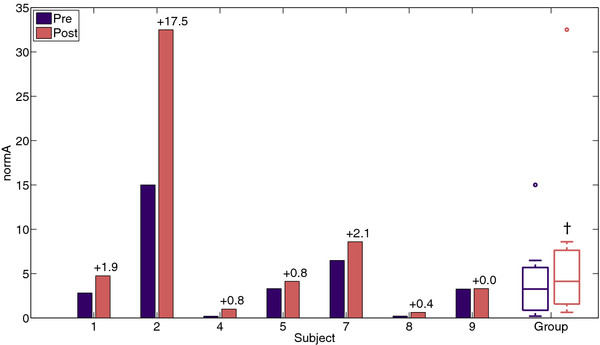
** Normalized circle area (normA) per subject and group mean before (Pre) and after (Post) training.** Changes in normA are displayed above the bars. † Indicates a statistically significant change.

After training all subjects were able to increase their normalized circle area, see Figure 6. Median normalized circle area increased from 3.3 (IQR: 1.5 – 4.9) % before training to 4.1 (IQR 2.1 – 6.7) % after training, which is statistically significant, p = 0.018.

**Figure 7:**
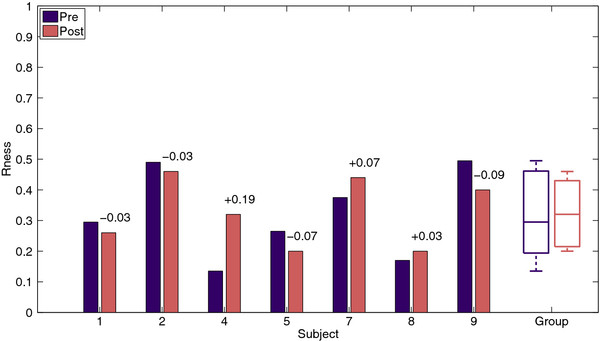
Roundness (Rness) of the circles drawn before (Pre) and after (Post) training.

Circle roundness did not change significantly, p = 1.0. After training, three subjects showed minor increases in circle roundness, whereas minor decreases in circle roundness were observed in four subjects. Median circle roundness was 0.30 (IQR: 0.22 – 0.43) during baseline measurements and 0.32 (IQR: 0.23 – 0.42) during the evaluation measurement. Changes in circle roundness are graphically displayed in Figure 7.

### Joint excursions

**Figure 8:**
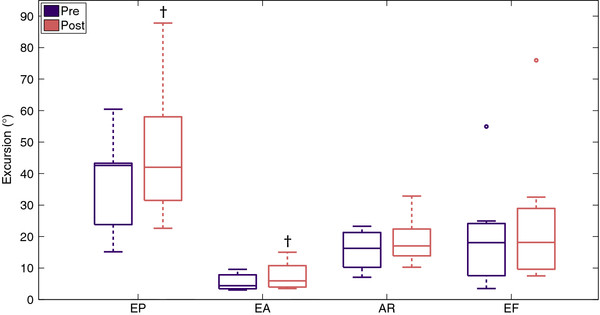
** Joint excursions during circle drawing before (Pre) and after (Post) training.** EP = Elevation Plane, EA = Elevation Angle, AR = Axial Rotation, EF = Elbow Flexion/extension. † Indicates a statistically significant change.

Joint excursions of EA, EP, AR and EF before (pre) and after (post) training are graphically displayed in Figure 8. After training five subjects increased the range of EP and two subjects (S5 and S9) had similar excursions during baseline and evaluation measurements. On group level, median increase in EP was 7.5 (IQR: 3.1 – 13.9)° which was a statistically significant (p = 0.043) change.

Excursions in EA were bigger in six subjects after training compared to baseline values. One subjects (S8) had similar values of EA during baseline and evaluation measurements. On group level a small but statistically significant (p = 0.028) increase of EA excursion from 4.4 (IQR: 3.6 – 7.3) ° to 5.9 (IQR: 4.1 – 10.1) ° was noticed.

Small decreases in AR excursions were observed in three subjects (S1, S5 and S9) whereas the remaining four subjects increased their AR excursions after training. Group median AR excursion increased from 16.2 (IQR: 11.9 – 20.5) ° before training to 17.0 (IQR: 13.9 – 22.2) ° after training. This increase was not statistically significant, p = 0.237.

Six subjects increased EF excursion after training, whereas one subject showed a small decrease. Median EF excursion increased from 18.0 (IQR: 8.8 – 23.3) ° before training to 18.1 (IQR: 10.6 – 25.4) ° after training. This increase was not statistically significant, p = 0.091. Maximal elbow extension (i.e. minimal EF, see Figure 3) increased in 5 out of 7 patients. The median change was −1.7 (IQR: -6.6 – -0.5)° which was not statistically significant, p = 0.128. Maximal elbow flexion increased in 5 out of 7 patients with a non significant (p = 0.398) median change of 1.1 (IQR: -0.6 – 4.2)°.

### Synergistic movement patterns

**Figure 9:**
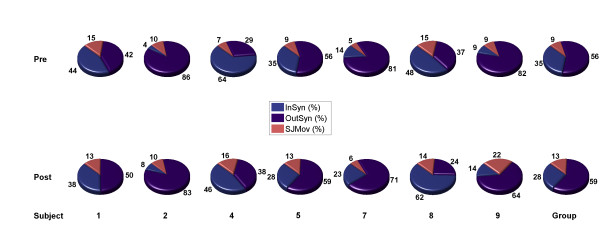
Occurence of synergistic movement patterns before (Pre) and after (Post) training.

The occurrence of synergistic movement patterns during circle drawing before and after training is graphically displayed in Figure 9. During baseline measurements median InSyn was 35.0 (IQR: 11.3 – 45.9) % of the movement time. Median OutSyn was 56.4 (IQR: 39.1 – 81.9) % of the movement time. During the remaining 8.7 (IQR: 7.9 – 12.3) % of the movement time at most one joint moved.

During evaluation measurements group median InSyn was 27.9 (IQR: 18.8 – 41.9) %, OutSyn 59.0 (IQR: 43.8 – 67.1) % and SJMov was 13.1 (IQR: 11.3 – 14.8) %. Changes in InSyn, OutSyn and SJMov were not statistically significant (p > 0.310).

### Correlation between training parameters and outcome measures

The strongest correlations were between the decrease in gravity compensation and change in FM (ρ = −0.54, p = 0.22) and change in normA (ρ = −0.61, p = 0.22). Training parameters such as the decrease of the level of gravity compensation and increase of training intensity were not significantly correlated to outcome measures (p ≥ 0.10). Between other outcome measures the only statistically significant correlation has been found between changes in EF and normA (ρ = 0.82, p = 0.023).

## Discussion

In this study changes in unsupported arm movements, induced by gravity compensation training, and the impact of abnormal coupling on arm movements in chronic stroke patients were studied by means of a circle drawing task [22]. After 6 weeks of moderately intense gravity compensated reach training, a sample of 7 chronic stroke patients showed improved arm function, as indicated by a median increase of 3 points on the FM scale. Subjects also significantly increased the active work area of the hand as indicated by the normalized circle area, whereas circle roundness remained almost constant. Statistically significant changes were observed in excursions of EP and EA, accompanied by increasing trends in excursions of AR and EF. The occurrence of synergistic movement patterns was similar before and after training.

The effect of abnormal coupling between shoulder and elbow movements on circle roundness was previously studied by Dipietro et al. [21]. Roundness of the circles drawn in the present study before training (mean ± SD 0.32 ± 0.14) was lower compared to the roundness of circles (mean 0.51) drawn in the study by Dipietro et al., despite a less severe patient group in the present study, as indicated by a higher FM score (FM = 27.1 and 20.5 respectively). This discrepancy is most likely related to the level of arm support during evaluation measurements. Dipietro et al. evaluated circle drawing while the subject’s arm was supported against gravity, while in the present study unsupported circle drawing was evaluated. Application of gravity compensation has been shown to reduce the influence of abnormal coupling between the shoulder and elbow [15,29] which is likely to result in rounder circles.

After robot-assisted, gravity compensated point-to-point reach training Dipietro et al. found an average increase in roundness of 0.10 [21,26]. The increase in roundness was the result of increasing minor axis of the fitted ellipse, while the major axis remained constant. In the present study, roundness remained similar before and after training, while the normalized circle area increased, i.e. both minor and major axes increased. A possible explanation for this discrepancy is a difference in training method. In both studies the arm was supported against gravity. However, in Dipietro et al. subjects who were not able to reach a target were assisted by the robot to complete the movement task, as well. Although recent reviews [30,31] that addressed technology supported arm training could not discern whether or not certain training modalities are more effective than others, it may be that differences in training modalities influenced roundness of the circles. A second explanation is related to the nature of the movement task that was assessed during evaluation measurements. In the study by Dipietro et al. subjects were asked to draw a copy of a template circle with a fixed radius of 14 cm. In the present study, subjects were asked to draw circles as big and as round as possible, during both evaluation measurements before and after the training period. As confirmed by the circle metrics, the focus of most subjects in the present study was in increasing circle area at the expense of increasing roundness.

Horizontal circle drawing can be seen as a continuous reaching task in the medio/lateral and forward/backward direction. Previous studies showed that gravity compensation training led to increased range of motion of the impaired arm as represented by increased maximal unsupported reach distance [13,18,19]. Ellis et al. [32] observed increased work area at various limb loadings after point-to-point reach training during which the level of arm support decreased progressively. Increased range of motion during unsupported arm movements was also found in the present study, as indicated by an increased normalized circle area after training.

Dipietro et al. [21] observed that the elliptical shapes drawn by stroke patients became rounder throughout the robot-assisted training period because the minor axis of the ellipse increased while the major axis of the ellipse remained almost constant. It was concluded that existing coupling between the shoulder and elbow joint remained after robot-aided reach training, but that the strength of the coupling decreased, which led to more selective control of the shoulder and elbow joint, as indicated by a lower correlation between shoulder horizontal ab-/adduction and elbow flexion/extension.

Although roundness and the occurrence of synergistic movement patterns were comparable before and after training in the present study, the changes in work area present some indications that a reduced impact of abnormal coupling may play a role in improved arm function. The improved ability to move the shoulder, in combination with a slight increase in elbow flexion and extension resulted in an increased circle area. However, when elbow extension is increased, i.e. the hand is moved away from the torso, higher muscle activations in the shoulder and elbow joint are needed to hold the arm against gravity, and stabilize the joints [33]. Consequently, these higher abduction torques will induce an increased amount of involuntary elbow flexion [9,10,13]. In other words, it is possible that stroke patients increased their work area because of a decreased impact of abnormal coupling between shoulder and elbow, but that the higher shoulder abduction torques needed to perform the movement task, again provoke an abnormal coupling at the elbow, resulting in similar amounts of movement within/out of synergistic patterns and consequently a proportional increase in both the major and minor axes of the fitted ellipse before and after training. A possibility to increase insight into the mechanisms involved in improving post stroke arm function, is to combine the circle drawing task used in the present study, in which patients maximize circle area, with a circle tracking task in which the size of the circles remains constant. With this second circle drawing task, the effect of synergistic movement patterns on circle shape can be studied, without the effects of increases in shoulder abduction torques that are needed to draw bigger circles. Differences in occurrence of synergistic movement patterns are likely to result in changes of circle area and roundness [21,22].

Besides involuntary coupling between the shoulder and elbow joint, many stroke patients also have to deal with muscle weakness [34] and/or strength imbalances across joints [35]. It is possible that patients strengthen their muscles during training, improve their temporal muscle activation or muscle coordination in general. To illustrate this, in the same sample of stroke patients, increased activity of agonist muscles during a maximal forward reaching task was observed after training [36]. This increased agonist muscle activity can be a result of increased ability to selectively activate agonist muscles, or improved control of agonist muscles. More research regarding changes in muscle activation patterns or changes in maximal voluntary torques (MVT) is needed to study the working mechanisms involved in changes of arm function after gravity compensated reach training.

### Limitations and recommendations

The present findings show that reach training with a low-cost arm support system and a low-tech computer game is able to improve hemiparetic arm function in a sample of chronic stroke patients. The reported increases in FM scores are comparable with interventions using more advanced training systems [30]. Nevertheless, results of the present study should be interpreted carefully, because of the small sample size of the study and the absence of a control group. Because of the small number of participants, it was not possible to subdivide the subjects into subgroups with different levels of stroke severity, to study potential differences in effects of gravity compensated reach training on hemiparetic arm function.

Since all subjects who participated were in the chronic phase after stroke, it may me be that subjects learned to avoid using the impaired arm [37]. It is not known whether improvement in hemiparetic arm function is due to improved neuromuscular control induced by the training, or by overcoming possible learned nonuse of the impaired arm. Inclusion of a control group in future research can yield information to what extent both processes occur.

Further research with larger and more homogeneous samples of stroke patients is needed to increase insight in the physiological mechanisms involved in the training induced changes in arm function, for example by studying training induced changes in muscle activation patterns.

### Clinical implications

The present study indicates that a moderately intense training program consisting of gravity-compensated point-to-point reach training within a VR augmented training environment can lead to increased work area of the hemiparetic arm in a sample of mildly to severely affected chronic stroke patients. Results concerning the underlying mechanisms causing these changes point towards a less pronounced influence of synergistic movement patterns, although more research is needed for further elucidation. The used training setup is simple and affordable and is therefore suitable to be deployed in clinical settings.

## Conclusions

Gravity compensated goal-directed reach training led to increased work area of the hemiparetic arm in a sample of 7 chronic stroke patients, as indicated by significantly increased normalized circle area. Circle roundness and the occurrence of synergistic movement patterns remained similar after the training period despite increased joint excursions of the shoulder and the elbow joints. A decreased strength of involuntary coupling between shoulder and elbow movements might play a role in increased arm function after gravity compensated reach training, but more research, specifically addressing muscle activation patterns, is needed to further elucidate the mechanisms involved in post stroke rehabilitation training. Inclusion of a circle tracking task besides the used maximal circle drawing task is helpful to study synergistic movement patterns in future research. Although training intensity was relatively low, improvement in arm function was achieved with the use of simple and affordable equipment that is suitable to deploy in clinical settings.

## Competing interests

The authors declare that they have no competing interests.

## Authors’ contributions

TK performed the design of the study, acquisition and analysis of data and drafting of the manuscript. BM assisted during acquisition of the data. AS developed and built the gravity compensation system. JR, JB and MJ were involved in interpretation of results and critical revision of the manuscript for important intellectual content. JB was also involved in conception and design of the study. GP was involved in design of the study, acquisition and interpretation of data, drafting of the manuscript and critical revision of the manuscript for important intellectual content. All authors have read and approved the final manuscript.
